# NAD^+^ Metabolism and Regulation: Lessons From Yeast

**DOI:** 10.3390/biom10020330

**Published:** 2020-02-19

**Authors:** Trevor Croft, Padmaja Venkatakrishnan, Su-Ju Lin

**Affiliations:** Department of Microbiology and Molecular Genetics, College of Biological Sciences, University of California, Davis, CA 95616, USA; tjcroft@ucdavis.edu (T.C.); pvenkatakrishnan@ucdavis.edu (P.V.)

**Keywords:** NAD^+^ metabolism, Sir2 family, nutrient signaling

## Abstract

Nicotinamide adenine dinucleotide (NAD^+^) is an essential metabolite involved in various cellular processes. The cellular NAD^+^ pool is maintained by three biosynthesis pathways, which are largely conserved from bacteria to human. NAD^+^ metabolism is an emerging therapeutic target for several human disorders including diabetes, cancer, and neuron degeneration. Factors regulating NAD^+^ homeostasis have remained incompletely understood due to the dynamic nature and complexity of NAD^+^ metabolism. Recent studies using the genetically tractable budding yeast *Saccharomyces cerevisiae* have identified novel NAD^+^ homeostasis factors. These findings help provide a molecular basis for how may NAD^+^ and NAD^+^ homeostasis factors contribute to the maintenance and regulation of cellular function. Here we summarize major NAD^+^ biosynthesis pathways, selected cellular processes that closely connect with and contribute to NAD^+^ homeostasis, and regulation of NAD^+^ metabolism by nutrient-sensing signaling pathways. We also extend the discussions to include possible implications of NAD^+^ homeostasis factors in human disorders. Understanding the cross-regulation and interconnections of NAD^+^ precursors and associated cellular pathways will help elucidate the mechanisms of the complex regulation of NAD^+^ homeostasis. These studies may also contribute to the development of effective NAD^+^-based therapeutic strategies specific for different types of NAD^+^ deficiency related disorders.

## 1. Introduction

NAD^+^, NADP^+^, and reduced equivalents NADH and NADPH are essential redox factors for many cellular enzymatic reactions. NAD^+^ also serves as a co-substrate in protein modifications, such as sirtuin (Sir2 family proteins)-mediated protein deacetylation and ADP-ribosylation. These modified proteins contribute to regulating Ca^2+^ signaling, chromatin structure, DNA repair, circadian rhythm, metabolic responses, and lifespan [1,2,3,4,5]. Several human diseases have been associated with aberrant NAD^+^ metabolism, including diabetes, cancer, and neuron degeneration [2,3,5,6,7,8,9,10,11,12]. Administration of NAD^+^ precursors such as nicotinamide (NAM), nicotinamide mononucleotide (NMN), nicotinic acid riboside (NaR), and nicotinamide riboside (NR) has been shown to increase NAD^+^ levels and ameliorate associated deficiencies in various model systems including human cells [3,5,6,7,8,9,10,11,13,14,15,16]. Nicotinic acid (NA) (niacin, vitamin B3) was first identified as a “Pellagra-preventive factor” in studies pioneered by Goldberger and Elvehjem in the early 1900s [17,18,19]. Later, NA was found to be involved in the biosynthesis of NAD^+^ [20,21]. Harden and Young first described the presence of NAD^+^ in cell-free fermentations of yeast juices in 1906 [22,23]. Later, von Euler and Warburg isolated pure fractions of NAD^+^ and NADP^+^, which were used to unravel the chemistry of these molecules [24,25,26].

Over many decades researchers have been unraveling the signaling pathways and cellular processes that contribute to the regulation of NAD^+^ and NAD^+^ precursor homeostasis; however, regulation of NAD^+^ metabolism and molecular mechanisms underlying NAD^+^ precursor treatments are still not completely understood. Studying NAD^+^ homeostasis is further complicated by the dynamic flexibility of precursors cells use to generate NAD^+^. For example, NAM is both an NAD^+^ precursor and an inhibitor of NAD^+^-dependent enzymes such as sirtuins [27,28]. Therefore NAM can modulate cellular function through pathways that depend on proper NAD^+^ homeostasis and sirtuin activity [29,30]. The precise roles of NAD^+^, sirtuins, and their downstream targets in diseases remain uncertain. Studying factors that contribute to the regulation of NAD^+^ homeostasis in budding yeast *Saccharomyces cerevisiae* may help shed some light on the role of NAD^+^ in disease. NAD^+^ biosynthesis is highly conserved between yeast and vertebrates. Employing the properties of yeast cells that constantly release and retrieve small NAD^+^ precursors [31,32,33], genetic tools have been developed to identify and study genes regulating NAD^+^ homeostasis. In yeast, mutants carrying single and multiple deletions of NAD^+^ pathway components and special defined growth conditions that pinpoint certain pathways are relatively easy to obtain. Several NAD^+^ homeostasis factors were uncovered in recent studies using NAD^+^ precursor-specific genetic screens [31,34,35,36]. Given the interconnections among NAD^+^ biosynthesis pathways and cellular processes, identification and studying additional NAD^+^ homeostasis factors are required to elucidate the regulation of cellular NAD^+^ metabolism. 

## 2. NAD^+^ Biosynthesis Pathways

**Figure 1:**
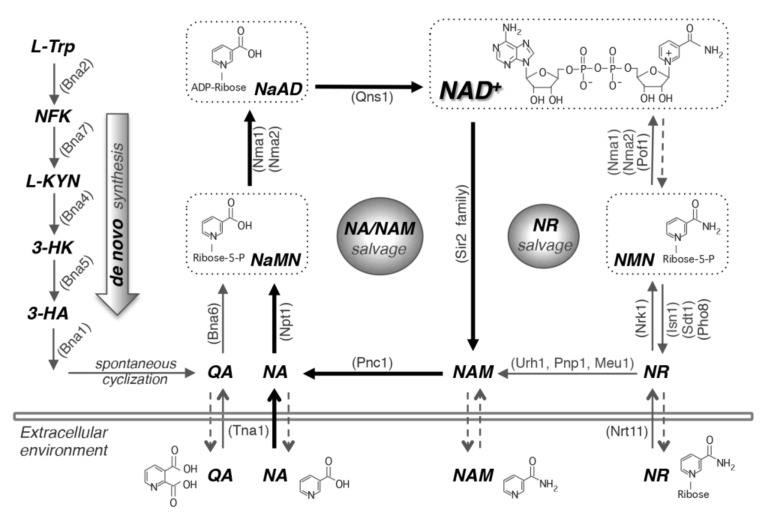
NAD^+^ biosynthesis pathways. In yeast cells, NAD^+^ can be made by salvaging precursors such as NA, NAM and NR or by de novo synthesis from tryptophan. Yeast cells also release and re-uptake these precursors. The de novo NAD^+^ synthesis (left panel) is mediated by Bna proteins (Bna2,7,4,5,1) leading to the production of NaMN. This pathway is inactive when NAD^+^ is abundant. The NA/NAM salvage pathway (center panel) also produces NaMN, which is then converted to NaAD and NAD^+^ by Nma1/2 and Qns1, respectively. NR salvage (right panel) connects to the NA/NAM salvage pathway by Urh1, Pnp1 and Meu1. NR turns into NMN by Nrk1, which is then converted to NAD^+^ by Nma1, Nma2 and Pof1. This model centers on NA/NAM salvage (highlighted with bold black arrows) because most yeast growth media contain abundant NA. Cells can also salvage NaR by converting it to NA or NaMN. For simplicity, NaR salvaging is not shown in this figure. Arrows with dashed lines indicate the mechanisms of these pathways remain unclear. NA, nicotinic acid. NAM, nicotinamide. NR, nicotinamide riboside. NaR, nicotinic acid riboside. QA, quinolinic acid. L-TRP, L-tryptophan. NFK, N-formylkynurenine. L-KYN, L-kynurenine. 3-HK, 3-hydroxykynurenine. 3-HA, 3-hydroxyanthranilic acid. NaMN, nicotinic acid mononucleotide. NaAD, deamido-NAD^+^. NMN, nicotinamide mononucleotide. Abbreviations of protein names are shown in parentheses. Bna2, tryptophan 2,3-dioxygenase. Bna7, kynurenine formamidase. Bna4, kynurenine 3-monooxygenase. Bna5, kynureninase. Bna1, 3-hydroxyanthranilate 3,4-dioxygenase. Bna6, quinolinic acid phosphoribosyltransferase. Nma1/2, NaMN/NMN adenylyltransferase. Qns1, glutamine-dependent NAD^+^ synthetase. Npt1, nicotinic acid phosphoribosyltransferase. Pnc1, nicotinamide deamidase. Sir2 family, NAD^+^-dependent protein deacetylases. Urh1, Pnp1 and Meu1, nucleosidases. Nrk1, NR kinase. Isn1 and Sdt1, nucleotidases. Pho8 and Pho5, phosphatases. Pof1, NMN adenylyltransferase. Tna1, NA and QA transporter. Nrt1, NR transporter.

NAD^+^ biosynthesis in yeast and humans is maintained by three pathways: de novo synthesis, NAM/NA salvage, and NR salvage (Figure 1). The NAD^+^ levels maintained by these pathways converge at several different points and consume cellular pools of ATP, phosphoribosyl pyrophosphate (PRPP), and glutamine while adding to total pools of ribose, AMP, phosphate, formate, alanine and glutamate. Some of these molecules contribute to other biosynthesis pathways or have signaling functions. Therefore, the cell must maintain these metabolites and their flux in a controlled manner. We do not fully understand all the mechanisms by which the cell can sense and tune these metabolites, but some known NAD^+^ homeostasis regulatory mechanisms include transcriptional control, feedback inhibition, nutrient sensing, and enzyme or metabolite compartmentalization [1,31,34,35,37,38,39,40,41,42]. 

The earliest indication of tryptophan contribution to NAD^+^ metabolism was in 1945 when Elvehjem supplemented tryptophan to rats fed a low NA corn diet and showed an increased level of NA [43]. The *de novo* pathway (also known as the kynurenine pathway) synthesizes NAD^+^ from tryptophan (Figure 1), spends the most cell resources, and is likely the least preferred pathway. This pathway is characterized by the synthesis of quinolinic acid (QA) from tryptophan by five enzymatic reactions by Bna proteins (Bna2, Bna7, Bna4, Bna5, Bna1) and a spontaneous cyclization (Figure 1) [44]. Bna6 then transfers the phosphoribose moiety of PRPP to QA, which produces nicotinic acid mononucleotide (NaMN), a molecule that is also produced by the NA/NAM salvage pathway. Dual specificity NaMN/NMN adenylyltransferases (Nmnats), Nma1 and Nma2 in yeast, are responsible for the conversion of NaMN to nicotinic acid adenine dinucleotide (NaAD) by the addition of the AMP moiety of ATP [45,46]. Amidation of NaAD to NAD^+^ is then carried out by the glutamine-dependent NAD^+^ synthetase Qns1 [47]. Several steps in the *de novo* pathway require molecular oxygen as a substrate (Bna2, Bna4 and Bna1) [44]. Therefore, cells grown under anaerobic conditions rely on the salvage pathways for NAD^+^ synthesis. When NAD^+^ is abundant, genes of the *de novo* specific pathway (*BNA* genes) are silenced by the sirtuin Hst1 [37]. Because Hst1 activity is dependent on NAD^+^, NAD^+^ depletion results in transcription activation of the de novo pathway. A recent study showed that the copper-sensing transcription factor Mac1 appeared to work in concert with Hst1 to repress *BNA* genes [34]. 

By 1958 Preiss and Handler had identified the pathway of NAD^+^ synthesis steps starting at NA, and this pathway is largely referred to as the Preiss–Handler pathway [48,49]. Unlike humans, in yeast this pathway also includes salvage of NAM and will be referred to as the NA/NAM salvage pathway (Figure 1). The NA/NAM salvage pathway produces NAD^+^ from precursors NA and NAM in yeast. Under NA abundant conditions, which describe most yeast growth media, NA/NAM salvage is the preferred NAD^+^ biosynthesis route and provides the cell with ample NAD^+^, high Hst1 activity and BNA (de novo pathway) gene repression [34,37,50]. In humans, NAM is converted to NMN by NAM phosphoribosyltransferase (Nampt) and then to NAD^+^ by the NR salvage branch, also first identified by Preiss and Handler in 1956 [51]. NAM is produced from many NAD^+^ consuming reactions including sirtuin mediated protein deacetylation and is also an inhibitor of these reactions [28,52,53,54]. Pnc1, a nicotinamidase found in budding yeast, hydrolyzes the amide group of NAM producing NA [55]. Deletion of PNC1 increases the concentration of NAM and inhibits sirtuin activity [39,40]. Like Bna6 of the de novo pathway, Npt1 produces NaMN by the transfer of the phosphoribose moiety of PRPP to NA. NAD^+^ biosynthesis from the NA/NAM salvage and de novo pathways converges at the formation of NaMN (Figure 1). 

Kornberg and Rowen were the first to identify NR as a precursor for NAD^+^ biosynthesis in 1951 [56]. With the exception of a few studies [57,58,59], NR-mediated NAD^+^ biosynthesis received little attention until 2004 when Brenner’s group reignited interest by demonstrating NR phosphorylation to NMN by NR kinase in yeast and human [60]. As mentioned above, in humans, Nampt also converts NAM to NMN, an enzyme not found in yeast. NR salvage can be considered the least expensive of the three pathways and requires no PRPP (Figure 1). However, NR can also be converted to NAM. In yeast this is accomplished by nucleosidases Urh1 and Pnp1 and redirects NR into NA/NAM salvage [16,61]. NR is phosphorylated by NR kinase, Nrk1, to produce NMN [60]. Nmnats (Nma1, Nma2 and Pof1 in yeast) are responsible for the conversion of NMN to NAD^+^ by the addition of the AMP moiety of ATP [36,45,46]. In yeast, the NR salvage branch confers flexibility that the other two pathways do not, which is in part due to compartmentalization of enzymes and precursors in this pathway. For instance, the vacuole plays an important role in the storage of NAD^+^-intermediate precursors, especially NR and NMN [35]. Fun26, an equilibrative transporter mediates transport of NR in and out of the vacuole. Pho8, a broad specificity vacuolar acid phosphatase, mediates the conversion of NMN to NR [1,35]. In addition, cytosolic nucleotidases Sdt1 and Isn1 convert NMN to NR [62]. Moreover, yeast cells release and re-uptake small NAD^+^ precursors such as NA, NAM, QA and NR (Figure 1) [31,36,63]. Specific transporters Tna1 (for NA and QA) [33,63] and Nrt1 (for NR) [64] are responsible for the uptake of NAD^+^ precursors whereas the mechanisms of precursor release remain unclear. Thus, the three branches of NAD^+^ biosynthesis are coordinated and provide the cell with NAD^+^ tuned to cellular requirements and environmental conditions. 

## 3. NAD^+^ and Its Derivatives in Redox Reactions

NAD(P)^+^ and NAD(P)H have long been recognized as coenzymes universally involved in oxidation-reduction reactions [24,25,26]. NAD(P)^+^ receives two electrons in the form of a hydride ion at position 4 of the NAM ring from a substrate reducing NAD(P)^+^ to NAD(P)H and release of a proton [65]. By coupling the oxidation of various molecules to the reduction of NAD(P)^+^, the cell temporarily stores energy in the form of high-energy electrons in the NAM ring of NAD(P)H. The electron arrangement of NAD(P)H is unfavorable, and the oxidation of NAD(P)H to NAD(P)^+^ is readily reversible. NADP^+^ has an additional phosphate group at the 2 position on the AMP moiety [66], which allows enzymes to distinguish NADP^+^ from NAD^+^. The stoichiometric concentrations of reduced and oxidized NAD(P)^+^ affords the cell to support the flow of electrons in two different directions. In catabolic pathways, where oxidation-reduction of NAD^+^ and NADH plays the dominant role, cell breakdown larger molecules to generate smaller building blocks and energy. High-energy electrons can be temporarily stored in the form of NADH and donated to the electron transport chain to make ATP via respiration [67,68]. NADP^+^ on the other hand is important for the reductive biosynthesis of molecules that help form the cell and protection from reactive oxygen species. Kornberg first showed that NADP(H) is produced from NAD(H) by NAD^+^ kinase in 1950 [69]. Yeast has two cytosolic NAD^+^ kinases that prefer NAD^+^ substrate producing NADP^+^ [70], and a mitochondrial NAD^+^ kinase that prefers NADH substrate producing NADPH [71]. NADPH is necessary to produce triacylglycerols, phospholipids, steroids, amino acids, and nucleotides [72]. NADPH is also required for anti-oxidative defense systems. For instance, it is involved in the direct reduction of inactive glutathione and thioredoxin to their active forms by glutathione and thioredoxin reductases [73,74,75].

## 4. Balancing the Redox State of NAD^+^ and NADH

**Figure 2:**
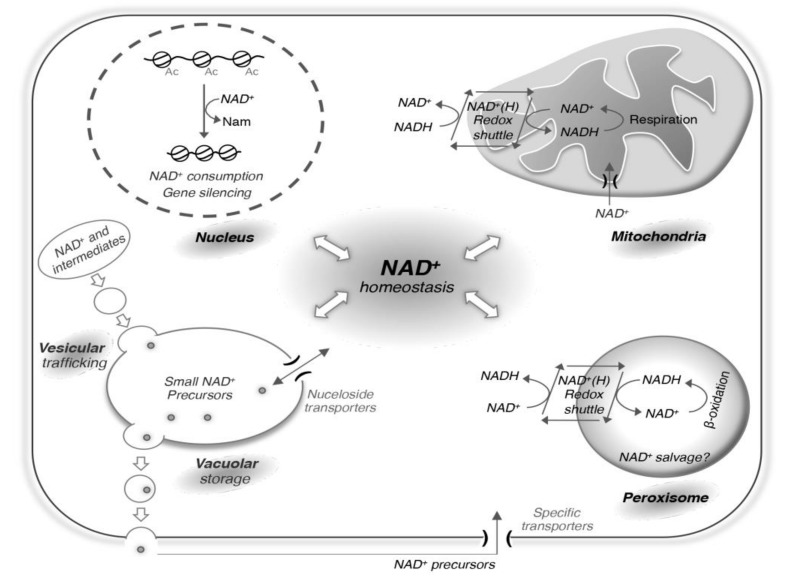
Cellular processes that are closely connected with NAD^+^ homeostasis. Various cellular processes together with compartmentalization of intracellular NAD^+^ and its derivatives contribute to the regulation of NAD^+^ homeostasis. For example, NAD^+^ and intermediates can enter the vacuole through vesicular tracking and are then converted to small NAD^+^ precursors. Small NAD^+^ precursors such as NR can travel between the vacuole and cytoplasm through specific nucleoside transporters. Small NAD^+^ precursors can exit cells likely by vesicular trafficking and re-enter through specific transporters on the plasma membrane. Sirtuin mediated gene silencing in the nucleus consumes NAD^+^. The nucleus and cytoplasm share the same NAD^+^ pool because NAD^+^ is anticipated to pass the nuclear pore by simple diffusion. The mitochondrial and peroxisomal NAD^+^(H) redox shuttle systems do not directly affect NAD^+^ metabolism, instead, they function to balance redox equivalents between the organellar and the cytosolic pools to regulate the NAD^+^/NADH ratio.

The redox state of the cell and the systems that the cell uses to balance this state largely depends on the growth conditions of the cell. For instance, cells grown aerobically have access to oxygen and can balance NADH by donating the electrons to the electron transport chain. Cells grown anaerobically only produce ATP by substrate-level phosphorylation and rely on other systems like fermentation to balance the NAD^+^/NADH ratio. In addition, because different pools (cytosolic and organelle) of NAD^+^ and NADH exist, redox equivalents are utilized to move back and forth from various compartments of the cell by use of shuttle systems. Here we discuss examples of such shuttle systems in mitochondria and peroxisomes (Figure 2), which also contribute to the maintenance of NAD^+^ homeostasis. In yeast, mitochondria do not synthesize their own NAD^+^, and rely on NAD^+^ transporters (Ndt1 and Ndt2) to maintain the NAD^+^ level [76]. Mitochondrial NAD^+^ kinases convert NAD^+^ to NADP^+^ or NADH to NADPH [71]. The portion of NAD^+^ that belongs to yeast mitochondria is unclear, but studies of other organisms suggest it can range from 20-85% of total cellular NAD^+^ [77]. Much less is known about how NAD^+^ and other derivatives are transported or made to support peroxisome pools. Several shuttle systems of mitochondria and peroxisomes have been identified, which include the malate-aspartate [78,79,80,81], ethanol-acetaldehyde [82,83,84], and the glycerol-3-phosphate shuttles [85,86,87,88,89,90]. These shuttle systems rely on dehydrogenases to oxidize or reduce substrates with reduction or oxidation of NAD(H). Therefore, there is no exchange in NAD^+^ or NADH between these pools, but rather an exchange of dehydrogenase products, which can either accept or donate the electrons in the adjacent pool of NAD(H). For example, respiration induced increase in NAD^+^/NADH ratio in the mitochondria can be transmitted to the cytosol by the malate-aspartate shuttle (Figure 2). Similarly, fatty acid β-oxidation induced decrease in NAD^+^/NADH ratio in peroxisomes can be balanced with the cytosolic pools by such shuttle systems. One interesting aspect of peroxisomes is they also contain nudix hydrolase Npy1 that hydrolyzes NADH to NMNH [36,91]. Its contribution to NAD^+^ homeostasis is unclear, but it could play an important role in either balancing redox state or removal of NAD^+^ metabolites from peroxisomes. Yeast also contains cytosol facing NADH dehydrogenases, Nde1 and Nde2, which donate electrons to the electron transport chain without transport of redox equivalents into mitochondria [92,93].

## 5. Cellular Processes that Consume NAD^+^

In addition to its redox role as described above, NAD^+^ is also consumed as a substrate. Consumption of NAD^+^ was noted early on by Mann and Quastel in 1941, who found that the consumption could be inhibited by NAM [94]. A year later Handler and Klein confirmed this finding and noted NAM was liberated by the reaction [95]. We now understand NAD^+^ has important non-redox roles in the modification of proteins and RNA. In yeast this is limited to deacetylation of proteins and 5’ capping of RNA [96,97]. However, in mammals this is expanded to the mono- and poly- addition of the ADP-ribose moiety of NAD^+^ to proteins in a process called ADP-ribosylation and carried out by a class of enzymes called poly ADP-ribose polymerases (PARPs). PARP activity has been linked to cell survival and genome stability [98]. 

Sirtuins are a class of enzymes highly conserved from yeast to human and involved in the deacetylation of proteins. Sirtuins transfer the acetyl group of a protein to the ADP-ribose moiety of NAD^+^ producing a deacetylated protein, o-acetyl-ADP-ribose and NAM. Budding yeast has five sirtuins (Sir2, Hst1-4) whereas humans have seven (SIRT1-7). The NAD^+^-dependent deacetylase activity was first identified for yeast Sir2, which is important for the silencing of the mating type loci, the ribosomal DNA genes and the subtelomeric regions by deacetylation of histones [99,100,101,102]. Sirtuins also have non-histone targets including metabolic enzymes and transcription factors [103,104] and affect many cellular processes including transcription regulation, genomic stability and cellular lifespan [103,104,105]. NAD^+^ and NAM play an important role in the regulation of sirtuin activity and downstream events. Deficiency in NAD^+^ production by deletion of biosynthesis enzymes abolishes sirtuin-mediated silencing [106,107]. Since NAM can both replenish NAD^+^ pool and inhibit the activity of NAD^+^ consuming enzymes such as sirtuins [28,52,53,54], maintaining NAM homeostasis is critical for cell function. In addition to re-entering NAD^+^ synthesis pathways, NAM can be cleared by methylation by NAM methyltransferase [108,109,110]. 

In some cases, the RNA polymerase has been found to add NAD^+^ to the 5’ ends of RNAs through the use of NAD^+^ (instead of ATP) as an initiating nucleotide. This NAD^+^ serves in place of a typical N7 methyl guanosine cap. This modification takes place in both eukaryotes and prokaryotes [96,111,112,113]. These NAD^+^-capped RNAs are inefficiently translated and less stable than N7 methyl guanosine capped RNAs [113]. It is thought that NAD^+^ competes with ATP for incorporation into the RNA in the first place, which raises many interesting questions on how concentrations of these metabolites and energy metabolism affect RNAs and downstream processes like translation. Additionally, some enzymes degrade NAD^+^ without modification of proteins or RNAs. In yeast, these include NUDIX hydrolases [36,91]. For instance, the previously mentioned NUDIX hydrolase Npy1 of peroxisomes cleaves NADH, producing NMNH and AMP. In human cells, SARM1 and CD38 cleave NAD^+^, and are believed to be contributors to various metabolic disorders and diseases [114,115,116].

## 6. Regulation of NAD^+^ Homeostasis

**Figure 3:**
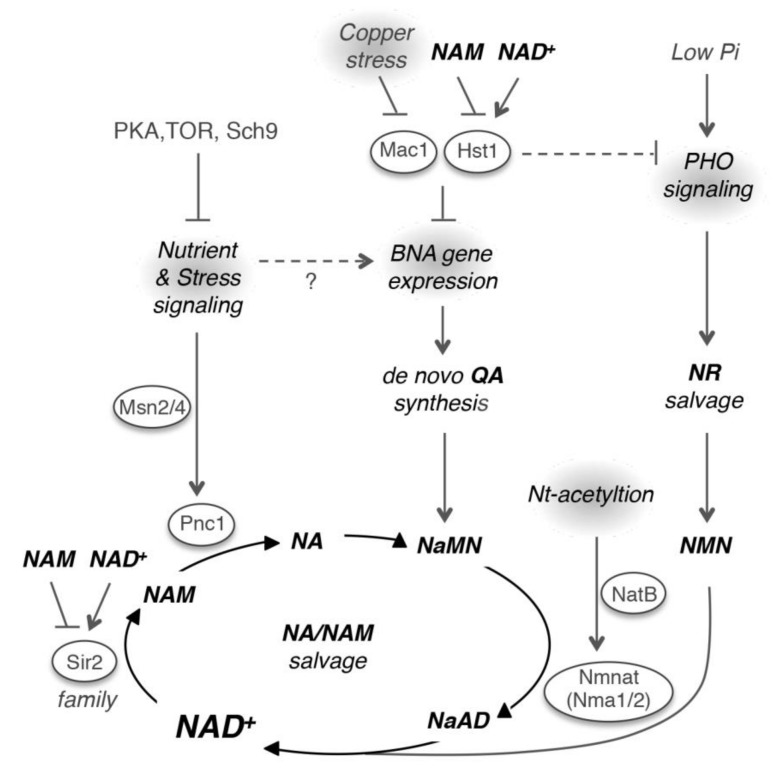
Regulation of NAD^+^ metabolism in yeast. A model depicting the regulation of NAD^+^ metabolism by cellular signaling pathways. NAD^+^ and NAD^+^ precursors are italicized and shown in bold. Abbreviations of protein names are highlighted by oval shapes. Dashed lines indicate additional evidence is required to reveal the mechanisms.

Studying yeast mutants with abnormal levels of specific NAD^+^ precursor(s) has led to the identification of novel NAD^+^ homeostasis factors [31,34,35,36]. These mutants were identified based on the observations that yeast cells constantly release and reuptake small NAD^+^ precursors [32,33]. For example, using a NAM release-specific reporter system, the subunits of the Nt-acetyltransferase NatB complex, Nat3 (catalytic subunit) and Mdm20 (regulatory subunit), were identified as potential regulators of NAD^+^ biosynthesis [31]. NatB mediated Nt-acetylation appears to be critical for maintaining proper Nmnat protein levels (Figure 3). In yeast, Nmnats are rate-limiting for NAD^+^-biosynthesis [31,42]. They participate in all three NAD^+^ biosynthesis pathways and are responsible for the conversion of NaMN to NaAD (de novo and NA/NAM salvage) and NMN to NAD^+^ (NR salvage) (Figure 1). Absence of Nt-acetylation led to an approximate 50% reduction in Nmnat proteins and NAD^+^ levels. Nt-acetylation is primarily a co-translational protein modification, which may alter protein folding, complex formation, localization, and degradation [117]. The precise roles of Nt-acetylation in Nmnats protein homeostasis and NAD^+^ biosynthesis have yet to be determined. 

Many mutants that scored high in the NA/NAM release screen had altered mitochondrial function, suggesting a link between mitochondrial function and the NA/NAM salvage pathway. The precise roles of mitochondria in NAM salvage and NAD^+^ homeostasis remain to be studied. Dysfunctional mitochondria need to be cleared from the cell and are targeted for degradation by bulk autophagy or selective mitophagy, which destroys mitochondria and its components. For instance, temperature sensitive ATP synthase mutants selectively degrade mitochondria by mitophagy at non-permissive temperature [118]. It is possible that a fraction of NAD^+^ is salvaged during this process. Another interesting finding in the NatB mutant was that increased NAM appeared to originate from vacuolar NR. Induction of autophagy by nitrogen starvation indeed increased the NAM and NR pools [31], which were diminished by deleting *ATG14*, an autophagy-specific factor [119,120]. Nitrogen starvation conditions closely mimicked the increased intracellular pools of NAM and NR in NatB mutant suggesting at least part of the increased NAM originates from increased autophagic degradation of upstream metabolites like NAD^+^ or NMN. Major sources may also include organelles that contain segregated pools of NAD^+^ or NAD^+^ intermediates, which are targeted by either bulk or selective autophagy. It would be interesting to understand the contribution of autophagic targeting of NAD^+^ and NAD^+^ intermediates to NAD^+^ homeostasis. 

In a separate study, a QA release-specific reporter system targeting the de novo branch was employed, which identified Mac1 as a novel NAD^+^ homeostasis factor [34]. Mac1 is a copper-sensing transcription factor that activates expression of copper transport genes in response to copper deprivation [121,122,123]. Cells lacking *MAC1* shared similar NAD^+^ phenotypes with the *hst1∆* mutant and deleting either *MAC1* or *HST1* was sufficient to abolish *BNA* gene repression [34]. It is suggested that Mac1 proteins may work with the Hst1-containing repressor complexes (consisting of Hst1, Rfm1, and Sum1) to repress *BNA* gene expression (Figure 3). Mechanisms of how Mac1 may function both as a transcription activator (for copper transport genes) and a co-repressor (for *BNA* genes) remain to be determined. When present at nutritional levels, copper binding to Mac1 results in a transcriptionally inactive state due to an intramolecular interaction. If cells are exposed to high-copper stresses, Mac1 is quickly degraded to prevent excess copper import [124,125,126]. Upon copper deprivation, Mac1 proteins become stable and can activate expression of copper transport genes. Therefore, it is suggested that under normal conditions, the transcriptionally inactive (copper-bound) form of Mac1 works with the Hst1 complex to represses *BNA* genes. These studies also suggest that both high and low copper stresses may impact de novo NAD^+^ biosynthesis via Mac1 and additional stress-signaling mechanisms. Given the close connection between copper homeostasis and mitochondrial respiration as well as the dependence of de novo NAD^+^ biosynthesis on O_2_, it might be beneficial if these processes are co-regulated. 

Several nutrient-sensing and stress signaling pathways have also been associated with NAD^+^ metabolism [34,127]. For example, activation of the low phosphate (Pi)-sensing *PHO* pathway has been associated with increased NR salvage activity (Figure 3) [35]. Interestingly, *PHO* activation was also observed in the low-NAD^+^ mutants, suggesting a cross-regulation between low-NAD^+^ and low-Pi signaling [35]. Moreover, the Pi moiety of NAD^+^ derivatives is a potential resource for Pi scavenging during Pi limitation. Interestingly, *PHO* activation was also observed in the *hst1∆* mutant [34] and cells with reduced amino acid sensing activity [128]. While the mechanisms remain unclear, coupling these pathways may be metabolically advantageous under certain conditions. Additional signaling pathways such as PKA (cyclic-AMP activated protein kinase A), Sch9 (yeast Akt), and TOR (target of rapamycin) may also play a role in NAD^+^ metabolism (Figure 3) [129]. These pathways appear to regulate stress response transcription factors, Msn2 and Msn4 [130,131,132], which have been shown to increase the expression of Pnc1 [38] in response to various mild stresses (Figure 3) [39]. 

The release and reuptake of NAD^+^ metabolites also contribute to NAD^+^ homeostasis. Yeast cells uptake NR, NA and QA (at low µM concentrations) via specific transporters Nrt1 (NR) and Tna1 (NA and QA) [33,63,64]. These NAD^+^ precursors may also enter cells by additional mechanisms when present at high levels. In yeast, high µM levels of NA can hinder the reuptake of released QA from the growth media [34] because QA and NA share the same transporter Tna1. Less is known about how NAD^+^ metabolites are released from yeast cells. It is suggested that both vesicular trafficking and vacuolar storage play a role (Figure 2) [1,31,133]. Vacuolar degradation of NAD^+^ intermediates appears to coincide with NAD^+^ salvage, and NAD^+^ metabolites may enter vacuole through vesicular transport and autophagy. NAD^+^ metabolites are then broken down into smaller precursors for storage or reuse. The equilibrative nucleoside transporter Fun26 (human hENT homolog) controls the balance of NR and likely other nucleosides between the vacuole and the cytoplasm [35]. Interestingly, although a vacuolar storage pool of NAM and NR has been observed, most excess QA is released extracellularly [33,34]. Together these studies demonstrate that compartmentalization of NAD^+^ metabolites also plays an important role in the regulation of NAD^+^ homeostasis.

## 7. NAD^+^ and Diseases

It has become apparent that altered NAD^+^ metabolism is correlated with several metabolic disorders and diseases, and therapeutic approaches by modulation of the NAD^+^ biosynthetic and consuming pathways using NAD^+^ precursors or chemical inhibitors are being actively explored [134,135,136,137]. Changes in NAD^+^ metabolism affect many cellular processes either through redox metabolism or activities of NAD^+^-consuming enzymes. It is often difficult to separate which aspects of NAD^+^ metabolism are important for different diseases, and it could likely be a combination of multiple factors. In addition, the rate-limiting NAD^+^ biosynthesis factors may differ in different cell types or under different environmental conditions. For example, Nampt is the rate-limiting factor for NAD^+^ synthesis in mammalian cells. Nampt has been implicated in several disease models in which both decreased and increased Nampt activities have been reported [134,138]. Nampt inhibition has been suggested to facilitate cancer cell killing because cancer cells have a higher demand for NAD^+^. In addition, because PARP mediated DNA repair requires NAD^+^, limiting NAD^+^ synthesis may also enhance cancer cell death [139]. Supporting this, the combination of PARP inhibitors and Nampt inhibitors was shown to induce synthetic lethality in breast cancer cells [140]. The roles of Nampt and NAD^+^ metabolism in metabolic diseases such as cancers and diabetes have been extensively discussed in several recent reviews [5,6,134,138,141,142,143]. Here we discuss the roles of NAD^+^, NAD^+^ biosynthesis enzymes Nmnats and NAD^+^-dependent sirtuins in select diseases of the nervous system. 

Dementia and diagnosable pathological alterations in the nervous system, which include demyelination and degeneration, occur in the late stages of Pellagra and possibly the first evidence that demonstrates the importance of NAD^+^ in the nervous system [144]. It became clearer that NAD^+^ has a role in disease of the nervous system from studies looking at Wallerian degeneration, which is characterized by the deterioration of axons upon cutting or crushing of a nerve. The discovery of the slow Wallerian degeneration mutant mouse (*Wld^s^*) immediately linked Nmnats with disease of the nervous system [145,146]. In *Wld^s^* mutant, Nmnat1 is fused at its N-terminus with the first 70 amino acids of UBE4B, a ubiquitin conjugation factor [147]. This change results in overexpression and redistribution of Nmnat1 to the cytoplasmic compartment of the axon. NAD^+^ biosynthesis in axon is normally supported by Nmnat2, but injury to the axon promotes the rapid degradation of Nmnat2 [148]. The *Wld^s^* fusion likely protects these axons through the synthesis of NAD^+^ as studies show it requires Nmnat1 enzymatic activity [147]. Additionally, expression of *NMNAT1* and *Wld^s^* seems to be protective in other neurodegenerative models [149,150,151]. Mutations in the *NMNAT1* gene are also responsible for inherited blindness of children in a disease called Leber congenital amaurosis, characterized by early onset and rapid progression of retinal degeneration [152,153,154,155]. While Nmnats have been implicated in various neurodegenerative diseases, the exact role they play is controversial and seems to include NAD^+^ biosynthesis and protein folding chaperone activity [151,156,157]. 

In support of the NAD^+^ synthesis model, overexpression of Nampt, the rate-limiting enzyme in human NAD^+^ biosynthesis, is also protective in some models. Recently SARM1, a toll-interleukin receptor like protein, was found to be important for a conserved axon death pathway [158,159]. Dimerization of SARM1 caused NAD^+^ depletion and consequently cell death [160], and eventually it was found that SARM1 cleaves NAD^+^ to ADP-ribose and NAM [115]. It is possible however to block SARM1 mediated axon degeneration through synthesis of NAD^+^ by overexpression of Nmnats or Nampt, or by supplementation with NR [160]. However, other pathways do not depend on the NAD^+^ biosynthesis activity of Nmnats. For instance, Nmnat2 activity is dispensable for preventing toxicity of Tau. It was shown that Nmnat2 complexes with Hsp90 to possibly promote refolding of toxic Tau [156]. Even in yeast models of neurodegenerative diseases, yeast Nmnats Nma1 and Nma2 provide protection from misfolded proteins [157]. Overall, these studies suggest increasing expression and slowing or prevention of degradation of Nmnats may provide a useful strategy for treating a variety of neurological diseases. 

Furthermore, depletion of NAD^+^ has a serious effect on NAD^+^ consuming enzymes such as sirtuins, which regulate several processes including mitochondria function and capacity to combat oxidative stress. In fact, mitochondria dysfunction and production of reactive oxygen species are closely associated with many neurodegenerative diseases [161,162,163,164,165,166,167,168]. For example, SIRT3 is localized to mitochondria and targets enzymes of various metabolic pathways including the TCA cycle, electron transport chain, and ketogenesis [169,170,171,172]. Importantly, it controls the production of reactive oxygen species by deacetylation and activation of antioxidant enzymes [173,174]. Cortical neurons in SIRT3 knockout mice are sensitive to excitatory, oxidative, and metabolic stress, and can be restored by SIRT3 gene delivery [175]. From the same study, using a Huntington’s disease model they found SIRT3 knockout mice quickly succumbed compared to mice with functional copies. In Alzheimer’s disease, SIRT3 protein levels are significantly decreased and promote dysfunction of mitochondria [176]. In some cases of Amyotrophic lateral sclerosis, mutations in superoxide dismutase SOD1 promote mitochondria dysfunction and fragmentation, and eventually motor neuron death [177]. Using mutant SOD1^G93A^ it has been shown that SIRT3 expression prevents mitochondria fragmentation and cell death [178]. In summary, various diseases of the nervous system appear to correlate with lower NAD^+^ levels or problems with NAD^+^-consuming and biosynthesis genes. Decreases in NAD^+^ and NAD^+^ homeostasis factors deregulate metabolism and sensitize the cell to reactive oxygen species. Future attempts to study these various diseases by promoting NAD^+^ synthesis seem warranted. Studies in yeast may help elucidate the interconnection between NAD^+^ metabolism and mitochondrial function. For example, many yeast mutants that release high NAM also show altered mitochondrial function [31]. Studying these mutants may provide some clues for more detailed mechanisms.

## 8. Conclusions and Perspectives

A balanced level of NAD^+^ is essential for maintaining proper cellular function. The cell has adapted many ways to regulate the biosynthesis of NAD^+^_,_ which include gene regulation, feedback inhibition, compartmentalization of enzymes and intermediate metabolites, and coordinates this biosynthesis via nutrient and energy sensing. While recent studies have identified novel regulators of NAD^+^ homeostasis, many questions remain unanswered. For example, in yeast, NAM can replenish NAD^+^ pools either by entering the NA/NAM salvage pathway or by de-repressing the de novo pathway **(**Figure 1) via inhibiting the activity of the NAD^+^-dependent sirtuin Hst1 [34,37]. It is unclear whether the de novo pathway in other organisms is also repressed by NAD^+^ and de-repressed by NAM in a sirtuin-dependent manner. Repression of de novo activity by NAD^+^ has been observed in bacteria [179,180]. However, NAM does not appear to de-repress de novo NAD^+^ synthesis activity in *E. coli* [179]. Since sirtuins are highly conserved across species [181,182], it will be informative to determine whether they also play a role in the regulation of de novo NAD^+^ biosynthesis in higher eukaryotes. Future studies to understand the multiple roles of NAD^+^ intermediates, as well as novel factors regulating NAD^+^ homeostasis are also highly anticipated

NAD^+^ metabolism is an emerging therapeutic target for several human diseases [5,7,11,14,183,184]. Supplementation of specific NAD^+^ precursors is often combined with genetic modifications and inhibitors of specific NAD^+^ biosynthesis steps to help channel the precursors to a more efficient NAD^+^ synthesis route [10,184,185]. Understanding the molecular basis and interconnection of multiple NAD^+^ metabolic pathways is therefore important for the development of disease-specific therapeutic strategies. It has been shown that these strategies are more effective if associated defects in specific NAD^+^ biosynthesis pathways/steps are known [10,11]. Moreover, specific NAD^+^ metabolites and NAD^+^ biosynthesis enzymes have also been reported to have additional functions [1,129]. For example, metabolites of the de novo pathway have been linked to several brain disorders [12,186]. Future studies to understand the multiple roles of NAD^+^ intermediates, as well as novel factors regulating NAD^+^ homeostasis are highly anticipated.
